# Efficacy of microsurgery for dural arteriovenous fistula: A systematic review protocol

**DOI:** 10.1097/MD.0000000000017288

**Published:** 2019-11-11

**Authors:** Guang-fu Song, He Wang, Xin Li, Chuan He, Ming-li Mao

**Affiliations:** a Department of Neurosurgery; b Department of Neurology, First Affiliated Hospital of Jiamusi University, Jiamusi; c Department of Neurosurgery, Beijing Xuanwu Hospital; d Department of Neurosurgery, Beijing Miyun District Hospital, Beijing, China.

**Keywords:** dural arteriovenous fistula, efficacy, microsurgery, safety, systematic review

## Abstract

**Background:**

Microsurgery is a treatment option for dural arteriovenous fistula (DAF), but its efficacy is still unclear. This study aims to assess the efficacy and safety of microsurgery for the treatment of patients with DAF.

**Methods:**

We will carry out this study assessing the use of microsurgery in patients with DAF from the following electronic databases: PUBMED, EMBASE, Cochrane Library, CINAHL, PsycINFO, Allied and Complementary Medicine Database, Chinese Biomedical Literature Database, and China National Knowledge Infrastructure. All those databases will be searched from inception to the present without language limitations. Two independent authors will perform study selection, data extraction, and methodological quality assessment. RevMan 5.3 Software will be applied for statistical analysis.

**Results:**

This study will assess the efficacy and safety of microsurgery for the treatment of patients with DAF through measuring initial treatment failure, late recurrence, neurological improvement, quality of life, and complications.

**Conclusion:**

This study will provide most recent evidence of microsurgery for the treatment of patients with DAF.

**Dissemination and ethics:**

The findings of this systematic review will be published in peer-reviewed journals. This systematic review dose not needs ethic approval, because it just analyzes the published data without individual information involvement.

**Systematic review registration:**

PROSPERO CRD42019144851.

## 1 Introduction

Dural arteriovenous fistula (DAF) is a very abnormal disorder, which directs connections between arteries and veins in the dura mater.^[1]^ It often occurs at the dura mater and its accessory tissues, such as the cerebral palsy and cerebellum, accounting for 10% to 15% of intracranial vascular malformations.^[2,3]^ This condition consists of 5 different types according to the position of the fistula. The highest incidence of DAF often occurred in the cavernous sinus area, accounting for 45.5%, followed by the areas of transverse sinus-sigmoid sinus, sacral, superior sagittal sinus, anterior cranial fossa, and the posterior fossa.^[4–7]^

A variety of clinical trials have reported that microsurgery can help to treat DAF effectively.^[8–19]^ Its efficacy and complications are, however, still inconclusive, and no systematic review has investigated this issue. Thus, this study will systematically explore the efficacy and safety of microsurgery for patients with DAF.

## 2 Methods and analysis

### 2.1 Eligibility criteria

#### 2.1.1 Participants/population

Patients with DAF, regardless the sex, age, and race will be included in this study.

#### 2.1.2 Interventions/exposure

In the experimental group, patients must receive microsurgery for the treatment.

In the control group, patients, however, can receive any treatments, except any types of microsurgery.

#### 2.1.3 Study types

All randomized controlled trials assessing the efficacy and safety of microsurgery for the treatment of DAF will be considered for inclusion.

#### 2.1.4 Outcome measurements

Outcome measurements consist of initial treatment failure, late recurrence, neurological improvement (as measured by National Institute of Health Stroke Scale score or other related scales), quality of life (as assessed by 36-Item Short Form Survey or other relevant tools), and complications.

### 2.2 Literature search

The current research collects and analyses studies on assessing the efficacy and safety of microsurgery for DAF from PUBMED, EMBASE, Cochrane Library, CINAHL, PsycINFO, Allied and Complementary Medicine Database, Chinese Biomedical Literature Database, and China National Knowledge Infrastructure. We will search all those databases from inception to the present without language restrictions. The example of search strategy for PUBMED is shown in Table 1. We will also apply similar search strategy to any other electronic databases.

**Table 1:**
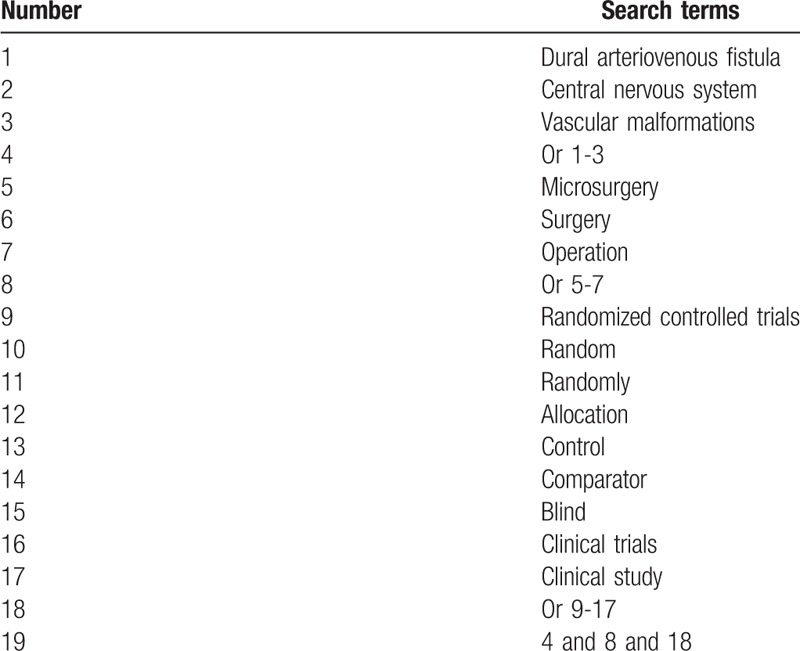
Search strategy applied in PUBMED database.

In addition, we will search any conference materials, dissertations, reports, and reference lists of relevant reviews.

### 2.3 Data selection

**Figure 1:**
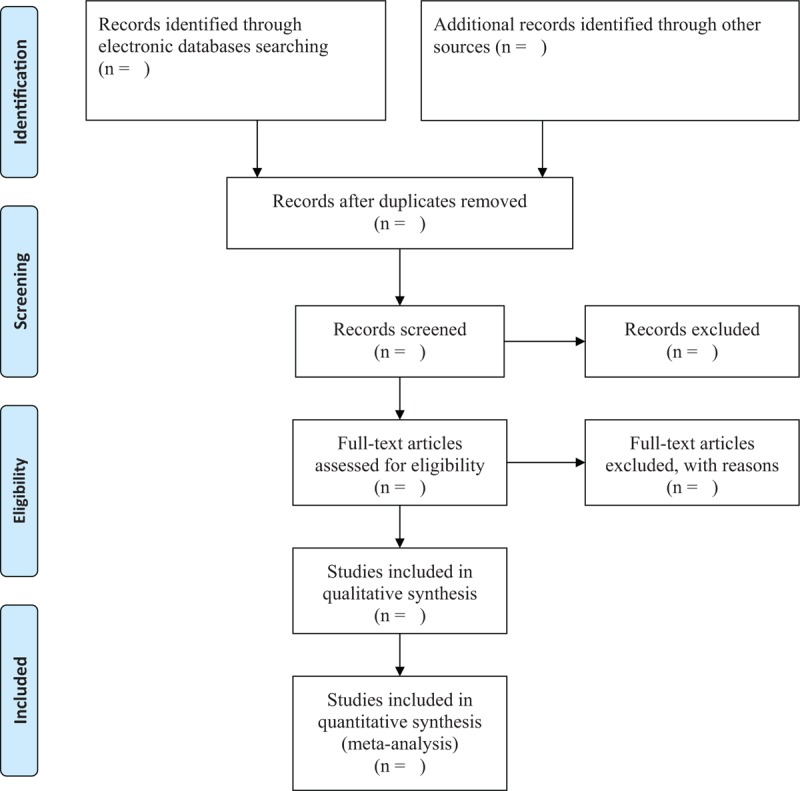
Flowchart of study selection.

Two independent authors will scrutinize the titles or abstracts firstly, and any irrelevant and duplicated studies will be excluded. Secondly, remaining studies will be carefully examined the full texts according to all eligibility criteria. The process of study selection will be presented in the flowchart in Figure 1. The reason for each study will be excluded at different stages. Any disagreements will be solved through discussion with the help of a third author.

### 2.4 Data extraction and management

Two authors will independently collect data from all eligible studies using previous data extracted sheet. Any different opinions between 2 authors will be solved by a third author. The extracted information comprises of characteristics of study (such as title, author, country, etc), sample size, study design, study methods, treatment details, outcome measurements, funding, and any other relevant information.

### 2.5 Dealing with missing data

If there is insufficient information or unclear data, we will contact original authors to request them. We will analyze the available data if these additional data cannot be achieved and we will discuss its impacts on the results of this study.

### 2.6 Risk of bias assessment

Two independent authors will utilize Cochrane risk of bias tool to assess the methodological quality for each eligible study. Any divergences between 2 authors will be settled down by a third author through discussion. This tool covers 7 domains, and each one is further classified as low, unclear, and high risk of bias.

### 2.7 Reporting bias assessment

Funnel plot and Egger regression test will be used to assess any possible reporting bias among eligible studies if >10 trials are included.

### 2.8 Assessment of heterogeneity

We will use *I*^2^ test to identify heterogeneity among eligible studies. If there is low heterogeneity (*I*^2^ ≤ 50%), a fixed-effect model will be applied for data pooling. On the contrary, if there is significant heterogeneity (*I*^2^ > 50%), a random-effect model will be used for data pooling.

### 2.9 Measurement of treatment effect

We will calculate the continuous data as mean difference or standardized mean difference and 95% confidence intervals (CIs), and dichotomous data as risk ratio and 95% CIs.

### 2.10 Statistical analysis

We will use RevMan 5.3 software to analyze all outcome data. According to the results of heterogeneity, we will pool the data using a fixed-effect model, and will carry out meta-analysis if *I*^2^ ≤50%. However, if *I*^2^ >50%, we will pool the data using a random-effect model and will conduct subgroup analysis at the same time. If there is still substantial heterogeneity after subgroup analysis, we will not perform meta-analysis, but will report outcome results with a narrative summary instead.

### 2.11 Subgroup analysis

Subgroup analysis will be performed based on the different forms of treatments, comparators, and outcome measurement tools.

### 2.12 Sensitivity analysis

We will conduct sensitivity analysis to identify the robustness of pooled outcomes by removing studies with high risk of bias.

## 3 Discussion

DAF is a rare disorder in the clinical practice. Although several studies have reported that microsurgery can help patients with DAF, no confirmed conclusion is made. Thus, this study firstly tries to investigate the efficacy and safety of microsurgery for patients with DAF. Its results may provide systematic and comprehensive assessment for the efficacy and safety of microsurgery for the patients with DAF. Such study will also provide help to make decisions regarding the future practice of microsurgery for DAF.

## Author contributions

**Conceptualization:** Guang-fu Song, Xin Li, Chuan He, Ming-li Mao.

**Data curation:** Guang-fu Song, He Wang, Chuan He, Ming-li Mao.

**Formal analysis:** Guang-fu Song, He Wang, Xin Li.

**Funding acquisition:** Ming-li Mao.

**Investigation:** Guang-fu Song, Ming-li Mao.

**Methodology:** Guang-fu Song, He Wang, Xin Li, Chuan He.

**Project administration:** Ming-li Mao.

**Resources:** Guang-fu Song, He Wang, Chuan He.

**Software:** He Wang, Xin Li, Chuan He.

**Supervision:** Ming-li Mao.

**Validation:** Guang-fu Song, Xin Li, Chuan He, Ming-li Mao.

**Visualization:** Guang-fu Song, He Wang, Ming-li Mao.

**Writing – original draft:** Guang-fu Song, He Wang, Xin Li, Chuan He, Ming-li Mao.

**Writing – review and editing:** Guang-fu Song, He Wang, Xin Li, Chuan He, Ming-li Mao.
